# Connectivity and free-surface effects in polymer glasses

**DOI:** 10.1038/s41598-019-40286-2

**Published:** 2019-03-07

**Authors:** Anna Lappala, Luke Sefton, Paul W. Fenimore, Eugene M. Terentjev

**Affiliations:** 1 Center for Nonlinear Studies, Los Alamos National Laboratory, Los Alamos, New Mexico USA; 2 Theoretical Biology and Biophysics, Los Alamos National Laboratory, Los Alamos, New Mexico USA; 3 Cavendish Laboratory, University of Cambridge, Cambridge, United Kingdom

## Abstract

The glass transition is one of the few unsolved problems in condensed matter physics: agreement on the cause of the slowing down of structural relaxation in glass-forming liquids is lacking. Glasses are amorphous solids, which do not possess the long-range crystalline order, yet display arrested dynamics and the shear elastic modulus characteristic of equilibrium elasticity. It has been suggested that due to the influence of intramolecular interactions and chain connectivity, the nature of the glass transition in polymers and in standard glass-formers is fundamentally different. Here, we discuss the role of connectivity in polymer glasses, demonstrating that although covalent bonding promotes glass formation, bonding sequentiality that defines a polymer chain is not critical in the bulk: glassy dynamics is purely a result of the number of connections per particle, independently of how these connections are formed, agreeing with the classical Phillips-Thorpe topological constraint theory. We show that bonding sequentiality does play an important role in the surface effects of the glass, highlighting a major difference between polymeric and colloidal glasses. Further, we identify the heterogenous dynamics of model coarse-grained polymer chains both in ‘bulk’ and near the free surface, and demonstrate characteristic domain patterns in local displacement and connectivity.

## Introduction

The glass transition has been a subject of extensive research since the late 1940s, and though many theories have been put forward, the problem still remains fundamentally unsolved, with an uncertainty as to whether the phenomenon is due to an underlying kinetic or a thermodynamic phase transition. During the transition from a liquid to a glass, particle dynamics can slow enormously over a small temperature range, despite very little structural difference at the atomic scale. This is because the particles become confined in ‘cages’ by their nearest neighbours, preventing free diffusion throughout the material. However, occasional movement over distances larger than of the particle diameter is possible by thermally activated ‘hopping’ processes, allowing single particles to escape from one cage to another. At high packing densities, this often requires cooperative motion of nearby particles, and several particles escape simultaneously^1^. Characteristic time scales arise: the short-time dynamics (*β*-relaxation) describes the movement of a particle within its cage of nearest neighbours, and the long-time dynamics (*α*-relaxation) is given by the cooperative relaxation of the cage confinement^2^.

There are major differences in the connectivity patterns between liquid-, crystalline- and glassy states. The average number of nearest neighbours in the first coordination shell is given by integrating the static radial distribution function *g*(*r*) up to its first minimum; this coordination number for central forces of the face-centered cubic (fcc) crystal is approximately z^*^ = 12, reflecting the optimal packing of hard spheres, and remains roughly constant as the temperature is lowered below the glass transition temperature *T*_g_^3^. In this manuscript, we will also refer to *direct contacts* that we define as monomers within 1.12σ (where σ is a dimensionless quantity that characterises distance), just below of the position of LJ potential minimum, making it easier to identify ‘touching’ particles. In Maxwell’s constraint counting method, systems with pairwise, spring-like forces acting between particles only become rigid above the isostatic connectivity threshold, where the number of constraints arising from such central force interactions just balances the number of internal degrees of freedom. According to Phillips-Thorpe, in a purely central force network, the Maxwell isostaticity value for sphere packing in three dimensions is z^*^ = 6, which is the marginal rigidity point at which glass transition takes place^4^. It is important to point out that this value is not applicable for finite-size systems that on average would have a lower coordination number 〈*z*〉^*^ < 6 as a result of surface effects, since there is a gradient associated with this value ranging from a high *z* in the centre of a collapsed globule and a low *z* closer to its surface^5^. Interestingly, according to Wang^6^ there is no conformational entropy contribution to the volume part of the free energy of a globule-it only becomes important in the interfacial region where there is density gradient.

Dynamical heterogeneity was first proposed in order to explain non-exponential relaxation patterns in single-component glassy systems, with different relaxation times contributing to produce the observed relaxation. Theoretical studies, experiments and simulations^7–10^ have since found evidence supporting the existence of spatially heterogeneous dynamics in a glassy state (for reviews of the experimental evidence for heterogeneous dynamics see^11,12^). Glotzer *et al*. quantified dynamical heterogeneity in simulations of supercooled glass forming liquids^13^ using cluster analysis, a scalar displacement correlation function and a four-point dynamical density correlation function and were able to identify mean cluster sizes, dynamical susceptibilities and characteristic time scales, which increase as *T*_g_ is approached from above. In addition, power law distributions of cluster sizes and “string-like” collective motions of particles were observed. Berthier *et al*. also found direct experimental evidence of a growing dynamic correlation length scale as the glass transition temperature is approached^14^.

**Figure 1:**
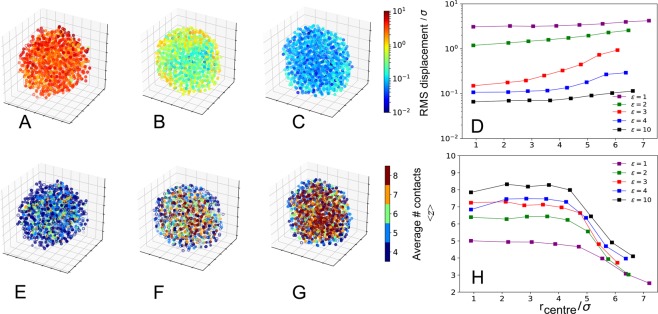
3D representations of the structure of globules with LJ interaction strengths of *ε* = 1 (**A**,**E**), *ε* = 3 (**B**,**F**) and *ε* = 10 (**C**,**G**) at time *t* = 3 × 10^5^*τ*. The particles are coloured according to displacement from the reference time *t* = 10^5^*τ* (**A**–**C**) and by the number of close contacts *z* (**E**–**G**). (**D**) The RMS displacement plotted as a function of radial distance *r*_centre_ outwards from the centre of mass for a variety of interaction strengths *ε*. Displacements are calculated at 1000*τ* after the time point of *t* = 3 × 10^5^*τ*– a frame at which the structure is fully collapsed and has reached a density plateau- and are averaged over all particles in each shell. (**H**) The average number of contacts per particle, 〈*z*〉, as a function of *r*_centre_ for varying *ε*.

Spatial heterogeneity is a result of local variation in the number of contacts and displacements. Here, we study both the contact density and the displacement of each monomer that was recorded from a given reference point- a collapsed state defined such that the radius of the collapsed globule is *R*_c_ ∝ *N*^1/3^ and the corresponding contact matrix is densely populated. We demonstrate glass formation under poor solvent conditions at high attraction strengths *ε* which is equivalent to simulating the demixing transition at low temperatures. To identify dynamically arrested particles within the collapsed globule (Fig. 1), we use the Maxwell rigidity condition, *z* ≥ 6, that directly allows us to identify the heterogeneity of the glassy state. One of the main challenges in studying the glass transition is the fact that identifying the arrested particles is non-trivial. Here, we define the dynamical arrest using two criteria: (1) Maxwell rigidity condition, and (2) relative displacement of a particle needs to be below a given threshold. Although the two criteria may seem to be mutually inclusive, this is not necessarily the case for polymer chains: one can identify particles with a low number of contacts that do not have high displacement–those are often found on the surface of the collapsed globule and the low displacement is due to connectivity–the bonding of particles links the surface particle to the particles inside the globule whose mobility is obstructed, resulting in an ‘apparent’ arrested state that cannot be distinguished from the actual arrested state due to crowding.

Understanding and regulating surface mobility is important in a wide range of materials^15–17^. According to Fakhraai and Forrest, surface mobility of polymer glasses is relevant for understanding adhesion, friction, and instabilities in thin polymer films and nanostructures used in lithography^18^. It is critical in a wide range of polymeric as well as non-polymeric glasses, and may have important consequences that have not been considered. According to Lam *et al*.^19^, the diverse penetration depths of surface effects and the peculiar properties of the inner-surface layer may be qualitatively applicable to different glassy systems where dynamics is dominated by collective motions of particles. The role of surface effects has important implications on the fabrication of polymer coatings designed to be responsive toward contact with small molecules such as drugs or sensors, as well as influence small molecule transport and diffusion^20^. Here, we study the complex relationship between surface effects and connectivity, shining light on how these phenomena regulate the collective motions in glassy dynamics and demonstrate that in the bulk, the same number of contacts leads to the same glass transition, irrespective of connectivity.

## Results

In order to study the effect of chain bonding on the glass transition, we used the widely accepted Kremer-Grest model for coarse-grained polymers^21^. Bonds between consecutive monomers are modeled by an attractive finitely extensible elastic potential, constructed by a combination of the attractive FENE part and the repulsive LJ part, while non-bonded particles interact through the LJ potential of variable attraction strength (see Methods below for detail). For the typical polymer parameters of Kremer and Grest, the bond potential is more tightly constrained and has a minimum at *r*_bond_ ~ 0.96*σ* (where *σ* is a dimensionless quantity that characterises distance), which is less than the pure LJ potential minimum at a universal *r*_min_ = 2^1/6^*σ*. As a result, there are two energetically preferred length scales, the bonded potential and the pairwise LJ interaction that are incommensurate. This disrupts regular packing and prevents the formation of a crystalline lattice^22,23^. The incompatibility of these length scales explains why this model is a good glass-former for polymer chains, but colloidal particles that interact solely through the LJ potential undergo spontaneous crystallisation. The two structures can be easily distinguished by observing their radial distribution functions (RDF)^24^. The colloidal crystal has many sharp peaks of high intensity that are indicative of regular close packing with long range order, while the polymer glass has a distribution function similar to a liquid, with a few peaks that are rapidly attenuated at increasing distance. For a flexible chain, if the position of the bond potential minimum is shifted such that *r*_bond_ = *r*_min_, the length scales become commensurate, and could then form a close-packed crystalline structure^25^. In a system of particles interacting through the LJ potential, introducing FENE-LJ bonds between consecutive particles with a competing length scale can be likened to introducing impurities into a crystal, and there is a critical number of bonds required to disrupt the lattice and prevent crystallisation, which we found to be ~14%.

**Figure 2:**
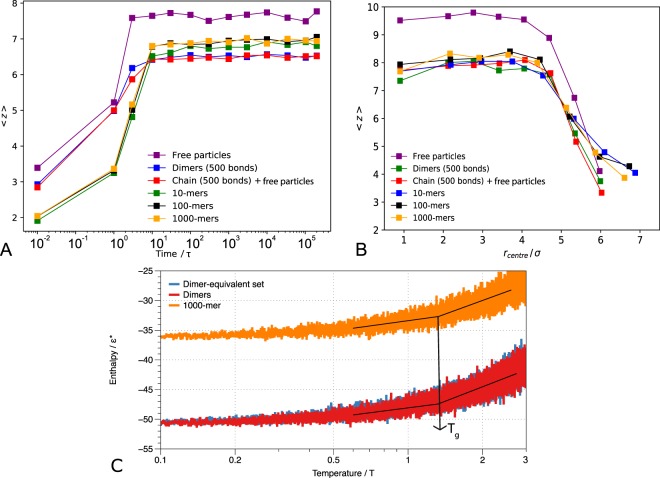
The average number of contacts 〈*z*〉 plotted (**A**) as a function of time after quenching for glassy globules (*ε* = 10) with different chain lengths. (**B**) as a function of radial distance *r*_centre_ outwards from the centre of mass for glassy globules (*ε* = 10) with a range of different chain lengths. Displacements are calculated at 1000*τ* after the reference time of *t* = 3 × 10^5^*τ*, (when the globule has achieved its compact state) and are averaged over all particles in each shell. (**C**) A plot of enthalpy (an equivalent of the total energy of the system) for glassy globules (ε = 10) as a function of temperature demonstrating the same *T*_g_ ≈ 1.5 for differently bonded systems (dimers, dimer-equivalent set and a single chain).

To demonstrate the effects of bonding along the chain, we study the time evolution of close contacts for non-bonded colloidal particles (*N* = 1000), comparing to those which are bonded as linear chains of different lengths Fig. 2 (for simulation set-up details, see Methods). To assess bonding sequentiality we compare a set of dimers, where only half of the particles are bonded, to a similar set-up where the number of bonds is the same (500), but the arrangement is such that 500 particles form a linearly connected chain, leaving the remaining 500 particles non-bonded; we call this set-up the dimer-equivalent set. In all cases, on quenching, 〈*z*〉 increases rapidly initially before reaching a plateau. There is a clear difference between the free particles and the bonded systems, which all behave very similarly and have consistently lower 〈*z*〉 than the free particles. However, the behaviour of the dimers and single chains with the same number of bonds as in the dimer system is more intriguing: initially, they behave similarly to the free particles and 〈*z*〉 increases as the globules condense rapidly, but 〈*z*〉 begins to plateau at a much lower value that is more similar to the equilibrium value for longer polymer chains. Moreover, the dimers and the single chain with half bonded and half free particles behave almost identically, despite the bonds being arranged in very different ways. This suggests that it is rather the number of bonds, and not the way in which they are arranged, that is important in determining the packing of the particles. From the radial distribution functions of the equilibrium states we observe that the crystal structure formed by the free particles is already disrupted by the 500 bonds present in the dimer- and dimer-equivalent sets.

The radial analysis shown in Fig. 1 for *ε* can be applied for varying chain lengths, as demonstrated in Fig. 2. Similar to the time evolution of 〈*z*〉, chains of varying lengths display near-identical behaviour, which differs from the free particles. The free particles have higher 〈*z*〉 in the ‘bulk’, but this falls more sharply and in fact becomes lower close to the surface. Curiously, the dimer- and dimer-equivalent sets behave like the longer chains in the ‘bulk’, but like the free particles close to the surface: this is due to the ‘apparent’ dynamical arrest that only takes place in longer chains, because the motions of the surface particles are constrained through bonded interactions.

To ensure that different connectivity patterns indeed share the same glass transition temperature *T*_g_, we performed a set of simulations at a heating rate of Γ_T_ = 10^−4^ from the collapsed glassy state (*ε* = 10) that was established from constant temperature (*T* = 1) simulations (the final structures of the constant temperature simulations served as the initial states for simulations with a temperature gradient). In Fig. 2C, we show that the system enthalpy as a function of temperature exhibits two linear regimes with different slopes corresponding to the high temperature liquid phase and in the low temperature solid phase. The glass transition temperature is defined by the intersection of the two lines.

In order to assess the mobility and connectivity of collapsed chains visualised in Fig. 1, we studied the displacement and contact density of individual monomers, highlighting the spatial distribution of contacts and displacement. Unsurprisingly, for the liquid globule (*ε* = 1), all particles interact weakly and therefore experience large displacements, leading to ergodic behaviour. For greater *ε*, however, the regions of highest displacement tend to be near the surface, while monomers with a large number of contacts tend to be close to the centre. This is an example of the surface effect, where the outer layers have a reduced glass transition temperature relative to the ‘bulk’, and therefore remain liquid while the ‘bulk’ becomes glassy^26^. Fig. 1 shows how the mean number of contacts 〈*z*〉 and the mean displacement of monomers changes with increasing radial distance from the centre of mass for a range of different LJ interaction strengths. As expected, 〈*z*〉 is greater for higher *ε* globules while the RMS displacement of particles is lower. This is because higher *ε* is equivalent to lower temperature and therefore more arrested–particles are held more strongly and experience caging, reducing their ability to move large distances. Furthermore, 〈*z*〉 is fairly constant close to the centre of all globules, suggesting that a large fraction of the globule is in the ‘bulk’ state, but decreases towards the edge of the globule, a clear demonstration of the surface effect for a system of finite size. For all globules, 〈*z*〉 begins to decrease at a radial distance of approximately 4.5*σ*, leading to the definition of the ‘bulk’ as all particles within 4.5*σ* of the centre of mass.

**Figure 3:**
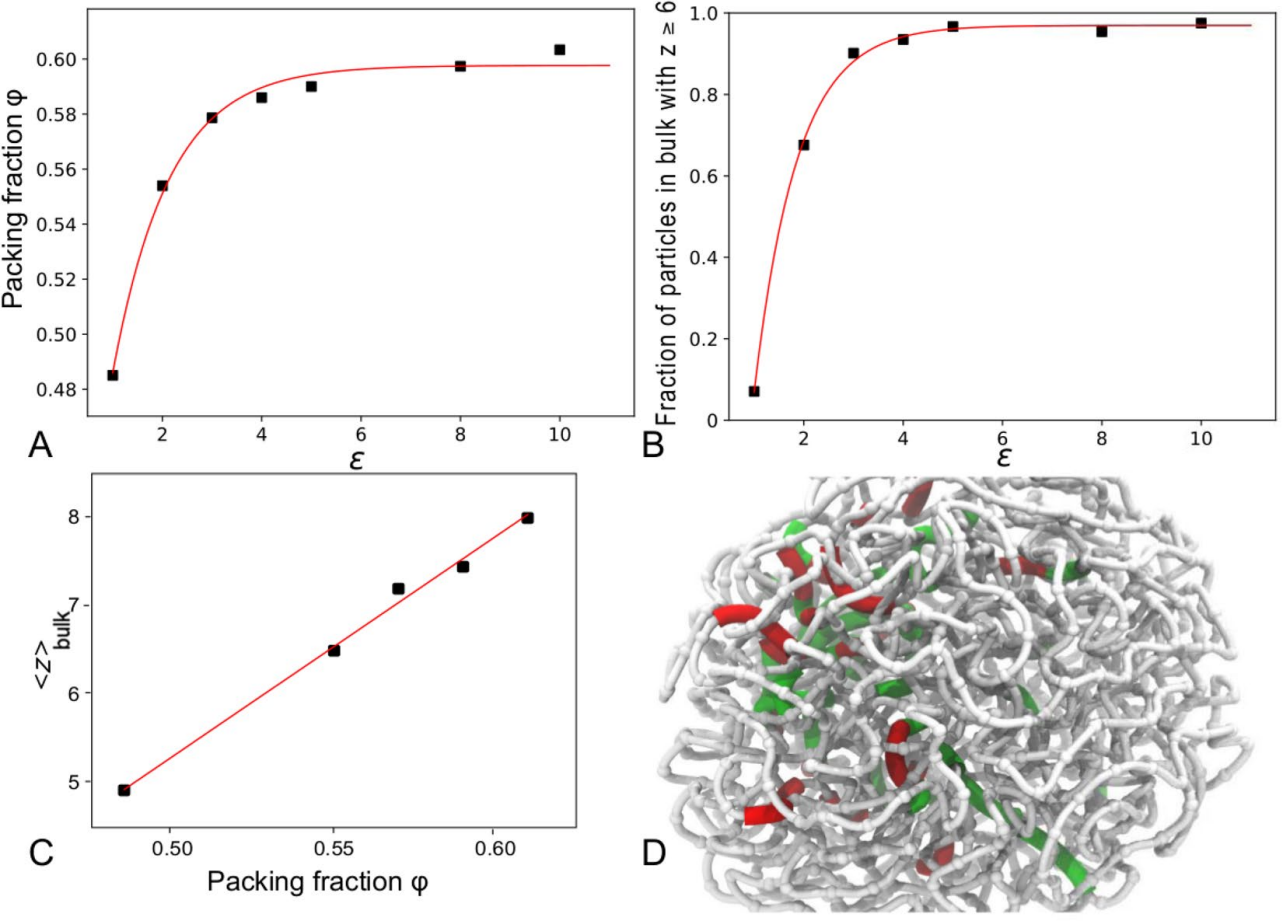
(**A**) The packing fraction of particles within the ‘bulk’ as a function of *ε*. (**B**) The fraction of particles that are glassy i.e that satisfy Maxwell’s central force marginal rigidity condition (*z* ≥ 6) for a range of *ε* values, with an exponential fit. (**C**) A linear relation between the number of contacts in the ‘bulk’ *z*_bulk_ and the packing fraction *ϕ*. (**D**) A 3D cubic spline-curve representation of a simulation snapshot of a single collapsed glassy *N* = 1000 chain with domains of high displacement (red) and low displacement (green) in the dynamically arrested ‘bulk’.

After establishing that 〈*z*〉 is fairly constant in the ‘bulk’, we evaluated the packing fraction in the ‘bulk’ of each globule for a range of *ε* values, as shown in Fig. 3A. The packing fraction increases sharply for small *ε* before beginning to plateau for large *ε* at a value of ~0.61. This is similar to the random close packing (RCP) fraction for spheres of ~0.64. In Fig. 3B we show the fraction of glassy particles with a number of contacts at or exceeding Maxwell’s isostaticity value (*z* ≥ z^*^ = 6) as a function of *ε* and fit an exponential function to the curves. These domains of glassy particles grow exponentially in the ‘bulk’ as *ε* increases, or equivalently as temperature decreases and the system undergoes glass transition, before reaching a plateau for high *ε* (low *T*).

**Figure 4:**
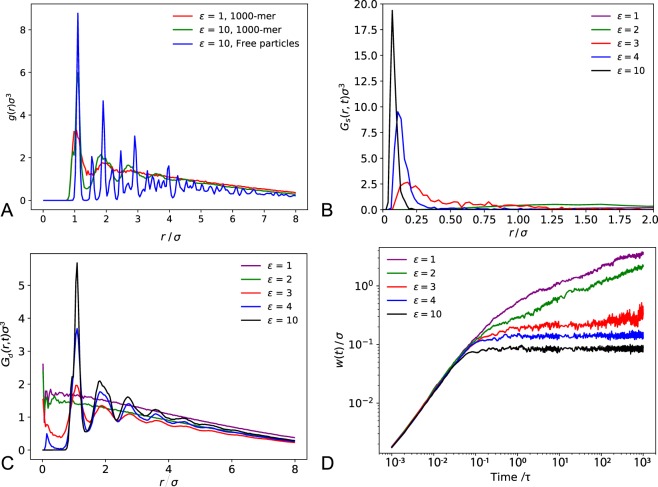
(**A**) The radial distribution function *g*(*r*) for a polymer liquid (*ε* = 1, 1000-mer), a polymer glass (*ε* = 10, 1000-mer) and a colloidal crystal (*ε* = 10, free particles). (**B**) The self part and (**C**) the distinct part of the van Hove correlation function plotted for different interaction strengths *ε* for a full 1000-mer chain. Values are calculated for the interval between 0.09*τ* and 100*τ* after the reference time of *t* = 3 × 10^5^*τ*, when the glassy states are fully established. (**D**) The FWHM *w*(*t*) of the self van Hove correlation function plotted as a function of time for a range of interaction strengths *ε*. Averages were calculated at increasing time after the reference *t* = 3 × 10^5^*τ*. Smaller time steps, 0.001*τ* were used here for higher resolution.

In order to emphasise the onset of caging dynamics in the glassy state, self- and distinct parts of the van Hove correlation function were evaluated for different *ε* at a long time (1000*τ*) after the reference of *t* = 3 × 10^5^*τ*, when the glassy states are in the arrested regime (Fig. 4). In the high-*ε* glassy state, the self-part peak is narrow and high intensity, indicating that the particles are confined to move over a small range close to their initial positions. As *ε* decreases, the peak width increases, which shows the particles become less strongly confined and are free to move larger distances. This is supported by the plot of the distinct part; at high *ε* it remains very similar to the RDF even after long times and there is almost zero probability of finding a different particle at a given position to the one that was there at *t* = 0, since the original particle is confined and still occupying the same position. However, as *ε* decreases, the peaks become less well-defined and the probability of finding a different particle in place of the original one increases until the distribution is almost linear. The reason that the distribution is not uniform and in fact decays with increasing *r* for the liquid globule (*ε* = 1), is due to the finite size of our systems–the diameter of each globule is only ~14*σ*. If one considers the locus of all points a distance *r* away from a given point in the globule, as *r* increases it is more likely that part of this locus lies outside the globule and therefore there is a reduced probability of finding a particle at a larger distance *r* from this given point. The time evolution of the FWHM *w*(*t*) for different *ε* is shown in Fig. 4. Initially, the particles move ballistically for all *ε*, as indicated by the slope of gradient ≈1. However, after just under 0.1*τ*, the behaviour diverges–the non-glassy globules (*ε* ≤ 2) become diffusive and *w*(*t*) continues to increase, while the dynamics in the glassy globules (*ε* ≥ 3) become arrested and *w*(*t*) reaches a plateau. Over long enough times one would expect to see jumps in *w*(*t*) due to hopping events. If not limited by the finite size of the globule, *w*(*t*) would continue to increase ∝*t*^0.5^ for the liquid globules, which would give a constant gradient of 0.5 in Fig. 4C. However, we instead see the gradient decreasing as *t* increases and *w*(*t*) tends towards an asymptote that is proportional to the radius of the globule.

## Discussion

To summarise, we demonstrated that while bonding between particles affects the structural organisation and the density of packing in polymer glasses, bonding sequentiality appears to be less relevant. However, the chain length plays a critical role in surface effects associated with glass transition–longer connected chains slow down the surface particles due to bonding, whereas the behaviour of shorter chains is dominated by Maxwell’s rigidity condition, and as the contact number decreases near the surface, the glass transition temperature is also reduced relative to the glassy core of the folded globule. Our findings may be extended to non-linear, branched or ring polymers, leading to a better understanding of the role of bonding patterns on the surface effects in non-linearly bonded polymers, resulting in tunable surface characteristics of soft materials, suggesting novel applications for soft- and biological material design. It would also be interesting to study the dependence of globule size on the effective depth of the surface layer and whether the characteristic density gradient is constant or system-size dependent.

## Methods

The LAMMPS simulation code was used to perform coarse-grained Langevin dynamics of Kremer-Grest model polymers^21,27^. Interactions between particles connected along the polymer chain were described via the FENE-LJ bond potential, which is a combination of the attractive FENE part^28^ and the repulsive part of the Lennard-Jones (LJ) potential cut off at *r* = 2^1/6^*σ*, the minimum of the LJ potential:

1 $${U}_{{\rm{FENE}}-{\rm{LJ}}}=-\,\frac{1}{2}\kappa {R}_{0}^{2}\,\mathrm{ln}\,[1-{(r/{R}_{0})}^{2}]+(\begin{array}{ll}4{\varepsilon }^{\ast }[{(\sigma /r)}^{12}-{(\sigma /r)}^{6}+{\varepsilon }^{\ast }], & r\le {2}^{1/6}\sigma \\ 0, & r > {2}^{1/6}\sigma \end{array}$$

where the careful analysis, re-used in numerous computational studies of polymers, gives the optimum parameter set of the maximum bond length *R*_0_ = 1.5*σ* and the spring constant *κ* = 30ε^*^/*σ*^2^ as in^21^. We choose the repulsive LJ strength ε^*^ = 1 in non-dimensional units) for this bond potential, which makes the equilibrium bond length *r*_bond_ = 0.96*σ*.

Non-consecutive particles interact via the full LJ potential, which is cut off at *r* = 3*σ* and has a variable depth of an attractive potential well *ε*:

2 $${U}_{{\rm{LJ}}}=(\begin{array}{ll}4\varepsilon [{(\sigma /r)}^{12}-{(\sigma /r)}^{6}+0.00137],\, & r\le 3\sigma \\ 0, & r > 3\sigma \end{array}$$

(the choice of constant is dictated by the condition that *U* → 0 at *r* → 3*σ*). The minimum of the LJ potential acting between non-covalently interacting monomers is at *r*_0_ = 2^1/6^*σ*, and we choose the radius of the shell representing monomers in direct contact as 1.12*σ*, just below of this position of LJ minimum.

**Figure 5:**
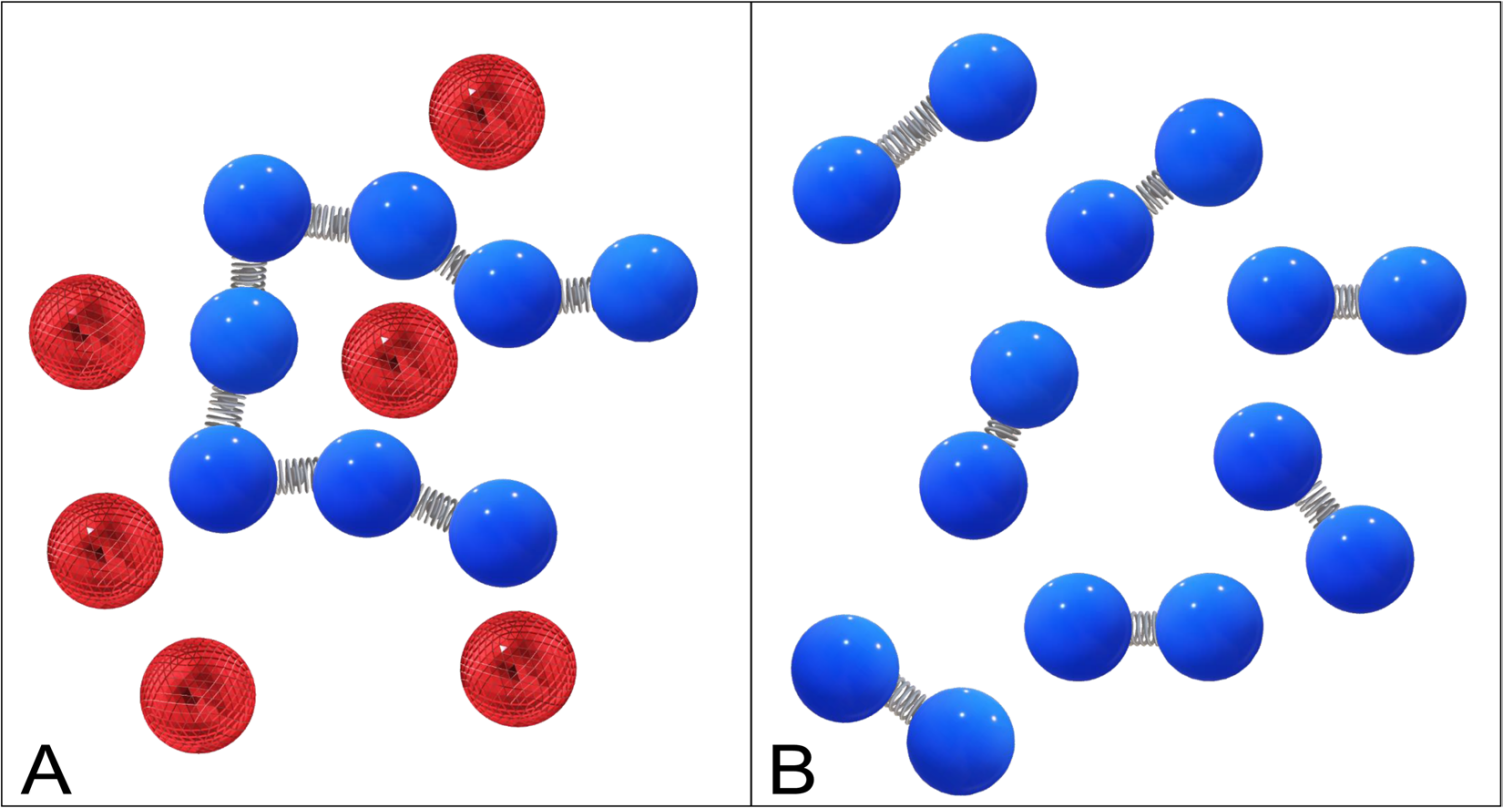
A pictorial representation comparing the dimers to the single chain with 500 bonds and 500 free particles (dimer-equivalent set), with bonds indicated by springs between particles.

Unless specifically stated otherwise, the average simulation temperature was controlled by the Langevin thermostat (simulation temperature was kept constant at *T*_start_ = *T*_end_ = 1, with the damping coefficient set to 1*τ*^−1^. Instead the LJ parameter *ε*, which is inversely proportional to temperature, was varied. Increasing *ε* from 1 to 10 is equivalent to lowering the temperature from *T* > *T*_g_ to *T* < *T*_g_, with *ε* = 1 corresponding to a liquid and *ε* = 10 to a glassy state. Timestep of 0.01*τ* was used (unless specified otherwise), where *τ* is the reduced (Lennard-Jones) time-a measure of how long it takes for the particle to move across its own size, defined as $\tau =\sigma \sqrt{m/u}$, where *m* is the characteristic mass, and *u* is the intrinsic energy of the system that is the same as parameter ε^*^ in the spring constant *κ*. Other physical parameters such as temperature and pressure are expressed via *u*^21,29^. The dense globular states of several systems were studied, all with the same number of particles (*N* = 1000):

1. Non-bonded particles interacting only via the LJ potential, effectively modelling colloidal particles.
2. Separate simulations with the same number of particles *N* = 1000, but varying the length of chains: 1000-mer, 100-mers, 10-mers and dimers.
3. A single chain with 500 bonds surrounded by 500 non-bonded particles “dimer-equivalent set” to compare to the dimer system with the same number of bonds, but arranged differently), as shown in Fig. 5.

The coil-globule transition occurs when a polymer is quenched i.e. collapsed when an attractive LJ interaction between all non-consecutive monomers is switched on at *t* = 0. The above systems were quenched in poor solvent to assess the role of connectivity during collapse. Depending on the depth of quenching (effectively measured by the depth of LJ potential well *ε*, while keeping the average temperature of the simulation constant), the expanded self-avoiding random walk rapidly collapses into a globule. The number of contacts between monomers was monitored, growing as a function of time as the globule becomes increasingly dense. In the expanded coil– the initial state of the simulations–only two covalent contacts per particle exist for a polymer chain, due to the linear connectivity along the chain. In the dense state, the number of physical (LJ) contacts increases. Since the total number of particles within a sphere *r*_min_ includes the particle itself, the average contact number is defined as *z* = (Ω − *N*)/*N*, where Ω is the total number of contacts in the whole system, thus excluding the self-contacts. All the initial states were equilibrated so that each particle has on average only two close contacts (its two consecutively bonded neighbours), corresponding to the open coil regime.

Having multiple polymer chains presents difficulties when trying to ensure that the structure condenses into one large globule, since separate chains are not bound together by bonds. To avoid this issue, we designed the following protocol:

1. Create monomer positions based on a self-avoiding walk.
2. Constrain all chains inside a sphere and gradually decrease its radius, while ensuring that the number of contacts per monomer is *z* ≤ 2, thus generating an open coil.
3. Equilibrate the system inside the sphere in good solvent (such that contact density remains *z* ≤ 2, i.e. an open coil) for *t* = 2000*τ*. This initial state was used for further studies at varying *ε*.
4. Remove confinement and switch on the attractive part of the LJ potential to imitate collapse in poor solvent, creating a collapsed globule.

It is important to point out that by enforcing confinement, one violates the early collapse kinetics of the system. However, this is not essential for the processes that are studied in this work and hence can be ignored.

**Figure 6:**
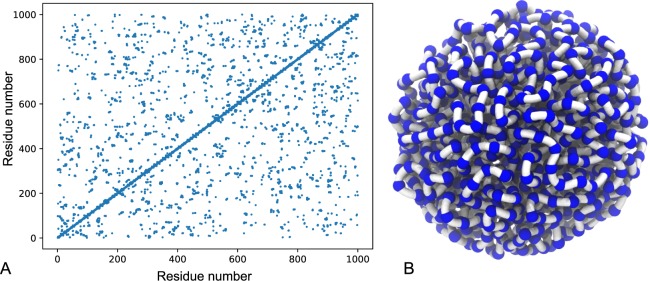
(**A**) an example contact map for a polymer glass globule (*ε* = 10 at *t* = 3 × 10^5^*τ*) along with (**B**) a 3D representation from a simulation of the corresponding globular structure.

The collapsed chains must be equilibrated for a suitable amount of time before measurements can be made; simply measuring when the potential energy of the system reaches a minimum is not sufficient, since there are often multiple configurations with the same minimum potential energy. The dynamics of a glass is very slow and the system can become ‘arrested’ in a local minimum for long periods of time before thermal fluctuations allow the system to overcome the barrier to a new minimum, leading to large simultaneous changes in morphology. To identify a collapsed state, one can refer to the established theoretical radius of a collapsed chain, *R*_c_ = *N*^1/3^. Contact maps (two-dimensional representations of the 3D connectivity) can be used to visualise the structure and morphology of folded chains and confirm that the chain is densely packed. For a given monomer, all other monomers that lie within a specified contact radius of that monomer are listed. The contacts are then represented in a two-dimensional plot, as shown in Fig. 6. There is always symmetry about the main diagonal, with the important structural features represented by off-diagonal elements. By observing the connectivity from contact maps and also 3D plots over time, one can easily identify any significant changes in morphology and determine a suitable equilibrium time, after which the structure remains very similar. All systems were equilibrated for *t* = 3 × 10^5^*τ* before making measurements.

We investigated the effect of the finite size of a globule by considering how the number of contacts and displacement of particles varies as a function of radial distance from the centre of mass *r*_centre_. The volume occupied by a collapsed globule can be divided into concentric spherical shells of thickness *dr*, centred on the centre of mass and each particle can be placed into a shell based on the position of its centre. For every particle, the number of contacts is calculated, as well as the displacement from its position at the chosen reference time. The average number of contacts and the RMS displacement is calculated across all particles within each shell. It is then instructive to consider the radial profile by plotting these quantities against the average radius of each corresponding shell. A suitable number of shells is chosen such that there is a sufficient number of particles in each shell to calculate reliable averages; eight shells were used for a system of 1000 particles. Using spherical shells is naturally most accurate for spherical globules where there is a constant angular distribution of particles for a given radius; non-spherical globules could introduce significant errors.

For a system of *N* particles, the van Hove correlation function is defined as the probability density of finding a particle *i* at position ***r*** at time *t*, given that there was a particle *j* at the origin at time *t* = 0, and can be written as:

3 $$G(r,t)=\frac{1}{N}\langle {\sum }_{i=1}^{N}{\sum }_{j=1}^{N}\delta (r-{r}_{i}(t)+{r}_{j}\mathrm{(0))}\rangle $$

where 〈⋅〉 represents an ensemble average and *δ*(⋅) is the three-dimensional Dirac delta function. At *t* = 0, this gives:

4 $$G(r,0)=\frac{1}{N}\langle {\sum }_{i=1}^{N}{\sum }_{j=1}^{N}\delta (r-{r}_{i}(0)+{r}_{j}(0))\rangle =\delta (r)+\rho g(r)$$

The van Hove correlation function can be separated into two parts: the “self” part *G*_s_(*r*, *t*) and the “distinct” part, *G*_d_(*r*, *t*). For non-zero times these can be written as follows:

5 $${G}_{s}(r,t)=\frac{1}{N}\langle {\sum }_{i=1}^{N}\delta (r-{r}_{i}(0)+{r}_{i}(t))\rangle ,\,{G}_{d}(r,t)=\frac{1}{N}\langle {\sum }_{i\ne j}^{N}\delta (r-{r}_{j}(0)+{r}_{j}(t))\rangle $$

One can obtain the self part at a given time *t* = *t*′ from a histogram of the displacements of all particles from their initial positions. *G*_s_(*r*, *t*) can be calculated by dividing the number of particles in each bin by the width of that bin, and then dividing by the total number of particles, *N*. The distinct part can be calculated in a very similar way to the radial distribution function, the only difference being that one calculates the distances from the position of each particle at time *t* = 0, rather than *t* = *t*′, to the positions of all other particles at *t* = *t*′.

**Figure 7:**
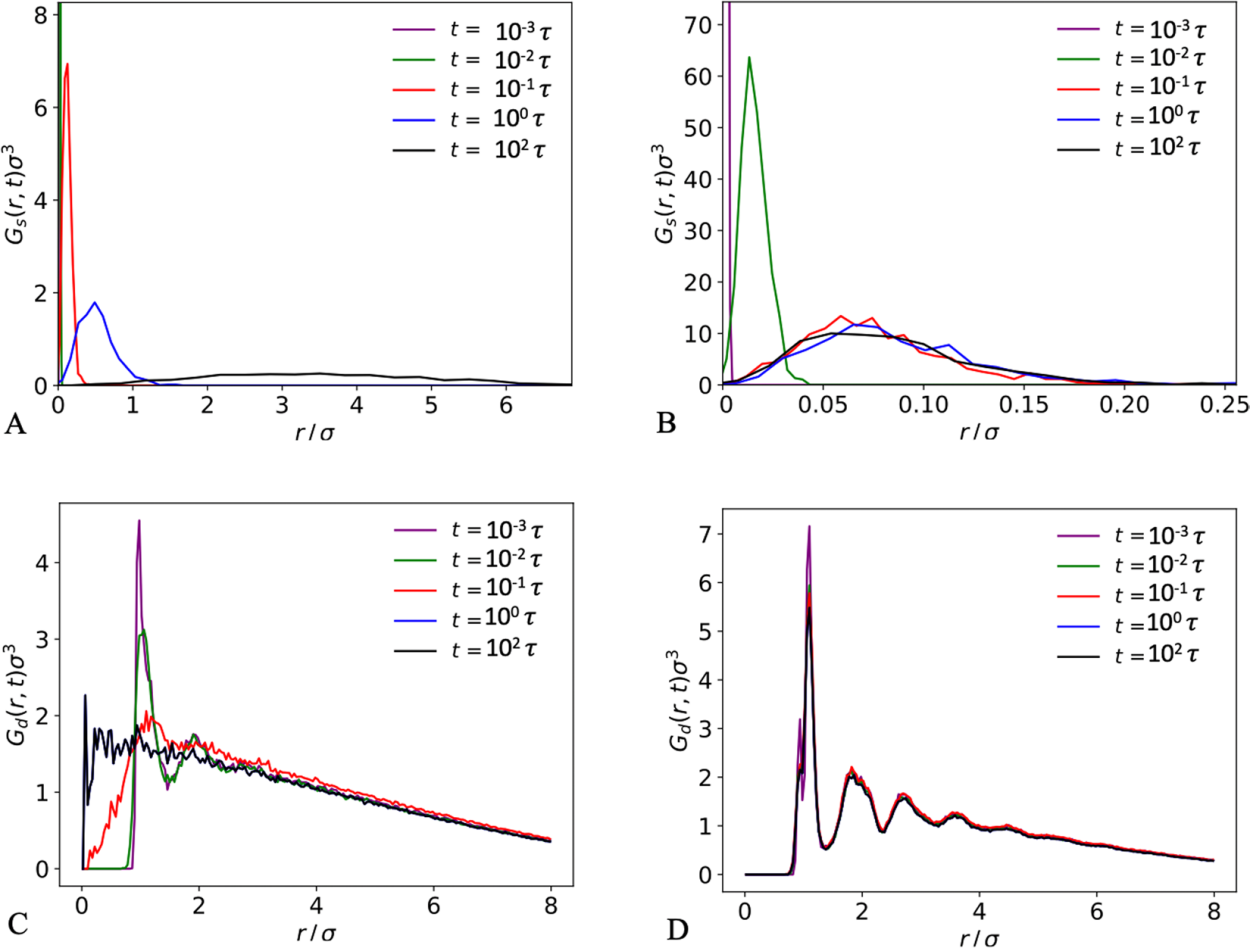
*G*_s_(*r*, *t*) and *G*_d_(*r*, *t*) plotted over a range of different *t* for (**A**,**C**) a liquid globule (*ε* = 1) and (**B**,**D**) a glassy globule (*ε* = 10). The *G*_s_(*r*, *t*) peaks at very small *t* are much narrower and more intense than at large *t*, so have been partially cut off in order to see the full range of behaviour.

The self and distinct parts of the van Hove correlation function were considered for different *ε* and chain lengths. One can characterise the range of particle movement, by defining the width *w*(*t*) of *G*_s_(*r*, *t*), which is equal to the full width at half maximum (FWHM) of the peak at time *t*. To reduce the effect of statistical noise, a Gaussian was fitted to each peak and the FWHM calculated from this Gaussian. At very small *t*, a large number of small bins is required to precisely define the sharp peak. At larger *t*, the displacement distribution peak broadens; using small bins with few particles in would mak e it difficult to fit an accurate Gaussian, so the bin size was increased with *t*. Starting from an equilibrated near-spherical globule at *t* = 3 × 10^5^*τ*, *G*_s_(*r*, *t*) and *w*(*t*) were calculated for a range of closely spaced times. In order to see the full range of behaviour, including the ballistic regime, the length of time step used was decreased by a factor of ten (0.001*τ*). Fig. 7 illustrates how *G*_s_(*r*, *t*) and *G*_d_(*r*, *t*) evolve over time for a liquid globule (*ε* = 1) and a glassy globule (*ε* = 10).
